# Nostalgia

**Published:** 1863-02

**Authors:** 


*Nostalgia.*—Surgeon D. C. Peters, in the *Medical Times*, gives the following picture of this remarkable disease:

That peculiar state of mind denominated nostalgia by medical writers, is a species of melancholy, or a mild type of insanity, caused by disappointment and a continuous longing for home. It is frequently aggravated by derangement of the stomach and bowels, and is daily met with in its worst form, in our military hospitals and prisons, and is especially marked in young subjects.

The symptoms produced by this aberration of the mind, are first, great mental dejection, loss of appetite, indifference to external influences, irregular action of the bowels, and slight hectic fever. As the disease progresses it is attended by hysterical weeping, a dull pain in the head, throbbing of the temporal arteries, anxious expression of the face, watchfulness, incontinence of urine, spermatorrhœa, increased hectic fever, and a general wasting of all the vital powers. The disease may terminate in resolution, or run on into cerebral derangement, typhoid fever, or any epidemic prevailing in the immediate vicinity, and frequently with fatal results. Among young prisoners of war it is the worst complication to be encountered, as the writer can truthfully affirm, after a few months' experience in treating several hundreds of these prisoners under the most favorable circumstances.

Fresh troops serving in the extreme South, where mail communications are irregular, and where the climate is very debilitating, suffer terribly this affection. The hospital of New Orleans and its vicinity, during the past summer, were filled with such cases, complicated with fevers and diarrhœa. The majority of them were young men from the Eastern States, whose love of home and kindred is a characteristic trait.

The diagnosis of nostalgia is not difficult in its early stages, although the patient may be unwilling to confess his mental weakness. It may possibly, however, be confounded with a depressed state of the mind, resulting from unexpected and sad intelligence.

The treatment of nostalgia would appear very simple, could we always at its onset remove the exciting cause, by allowing the patient the free exercise of his will; but from obvious reasons this is usually an impossibility. The strict rules, usages, and exigences of military service are insurmountable barriers against granting too free indulgence to soldiers. The surgeon must carefully attempt to relieve the patient's mind of its injurious burden by other means, such as kindness, free exercise, bathing, and agreeable associations, while he improves the tone of the stomach and bowels by generous diet and tonics. In cases where complications exist, notwithstanding his zealous efforts, the symptoms will frequently baffle his skill, and then as a *dernier resort*, and in order to save life, or prevent permanent disability, he must recommend the man's discharge from the service.

---

*Bromide of Potassium in Ovarian Tumor.*—Dr. P. Stewart. of Peekskill, N. Y., reports in the *Reporter* a case of trilobular ovarian tumor, each lobe two-thirds the size of a child's head at birth, successfully treated by Bromide of Potassium. Three grs. gradually increased to eight grs., were directed to be taken in Comp. Syrup Sarsaparilla three times a day—to be continued without interruption for a year. Improvement commenced in two months.

